# Supplementary material for: Compositional Data and Microbiota Analysis: Imagination and Reality
Source: Microorganisms. 2024 Jul 20;12(7):1484. doi: 10.3390/microorganisms12071484 (PMC11279367; doi:10.3390/microorganisms12071484)
Supplement: Supplementary file 1 [file microorganisms-12-01484-s001.zip › microorganisms-3075444-supplementary.pdf]

The data shown in Table S1–S3 are the artificial compositional data and assuming absolute amount of bacteria (counting data). Row names are numbered to distinguish each data item. A, B, C, D, E and F are column names and are used to distinguish columns.

Table S1. Compositional data showing relative abundance of bacteria A to F.

| ID | A | B | C | D | E | F | Total |
|---|---|---|---|---|---|---|---|
| 1 | 0.491848 | 0.412124 | 0.080724 | 0.012704 | 0.002201 | 0.0004 | 1 |
| 2 | 0.5956 | 0.3234 | 0.066 | 0.0134 | 0.0004 | 0.0012 | 1 |
| 3 | 0.4793 | 0.4603 | 0.0019 | 0.033 | 0.0239 | 0.0016 | 1 |
| 4 | 0.251899 | 0.481014 | 0.175099 | 0.068991 | 0.02289 | 0.000107 | 1 |
| 5 | 0.3284 | 0.6381 | 0.0237 | 0.0071 | 0.0004 | 0.0023 | 1 |
| 6 | 0.4632 | 0.1747 | 0.2209 | 0.1358 | 0.0049 | 0.0005 | 1 |
| 7 | 0.460347 | 0.389876 | 0.048644 | 0.097391 | 0.003326 | 0.000416 | 1 |
| 8 | 0.3645 | 0.4677 | 0.0099 | 0.063 | 0.0948 | 0.0001 | 1 |
| 9 | 0.520159 | 0.32316 | 0.102378 | 0.05369 | 0.000102 | 0.00051 | 1 |
| 10 | 0.1263 | 0.7057 | 0.0688 | 0.0779 | 0.0195 | 0.0018 | 1 |
| 11 | 0.408231 | 0.523948 | 0.056249 | 0.010968 | 0.000402 | 0.000201 | 1 |
| 12 | 0.4295 | 0.4122 | 0.0529 | 0.1028 | 0.0018 | 0.0008 | 1 |
| 13 | 0.362036 | 0.578558 | 0.027803 | 0.029103 | 0.0012 | 0.0013 | 1 |
| 14 | 0.379 | 0.4992 | 0.0387 | 0.0291 | 0.0537 | 0.0003 | 1 |
| 15 | 0.1935 | 0.6916 | 0.0007 | 0.0157 | 0.0983 | 0.0002 | 1 |
| 16 | 0.4286 | 0.5322 | 0.0096 | 0.0216 | 0.0079 | 0.0001 | 1 |
| 17 | 0.6134 | 0.318 | 0.0406 | 0.0277 | 0.0002 | 0.0001 | 1 |
| 18 | 0.2746 | 0.6937 | 0.0087 | 0.0217 | 0.0001 | 0.0012 | 1 |
| 19 | 0.2833 | 0.6104 | 0.0858 | 0.0187 | 0.0002 | 0.0016 | 1 |
| 20 | 0.3304 | 0.4132 | 0.0483 | 0.0372 | 0.1707 | 0.0002 | 1 |
| 21 | 0.438 | 0.4067 | 0.0766 | 0.0773 | 0.0013 | 0.0001 | 1 |
| 22 | 0.191219 | 0.717872 | 0.042804 | 0.034303 | 0.012501 | 0.0013 | 1 |
| 23 | 0.10281 | 0.588859 | 0.248925 | 0.032303 | 0.026903 | 0.0002 | 1 |
| 24 | 0.2561 | 0.3959 | 0.0361 | 0.0506 | 0.2609 | 0.0004 | 1 |
| 25 | 0.2924 | 0.5479 | 0.022 | 0.0426 | 0.0949 | 0.0002 | 1 |
| 26 | 0.2608 | 0.6638 | 0.0018 | 0.0377 | 0.0355 | 0.0004 | 1 |
| 27 | 0.287015 | 0.537915 | 0.111244 | 0.063125 | 0.0001 | 0.0006 | 1 |
| 28 | 0.4628 | 0.4616 | 0.0395 | 0.0331 | 0.0029 | 0.0001 | 1 |
| 29 | 0.4907 | 0.3577 | 0.0365 | 0.0681 | 0.0467 | 0.0003 | 1 |
| 30 | 0.4517 | 0.2474 | 0.0453 | 0.0422 | 0.2133 | 0.0001 | 1 |
| 31 | 0.354 | 0.547 | 0.0379 | 0.0378 | 0.0184 | 0.0049 | 1 |
| 32 | 0.668021 | 0.189718 | 0.129222 | 0.00513 | 0.003741 | 0.004168 | 1 |
| 33 | 0.3343 | 0.564 | 0.0394 | 0.0293 | 0.0327 | 0.0003 | 1 |
| 34 | 0.607384 | 0.362142 | 0.018617 | 0.010088 | 0.00052 | 0.001248 | 1 |
| 35 | 0.390823 | 0.463912 | 0.092376 | 0.052177 | 0.000102 | 0.000609 | 1 |
| 36 | 0.4583 | 0.3956 | 0.0766 | 0.0228 | 0.0466 | 0.0001 | 1 |
| 37 | 0.3969 | 0.5351 | 0.0433 | 0.0232 | 0.0002 | 0.0013 | 1 |
| 38 | 0.3287 | 0.3145 | 0.3319 | 0.0234 | 0.0007 | 0.0008 | 1 |
| 39 | 0.374459 | 0.483533 | 0.02679 | 0.115017 | 0.000101 | 0.000101 | 1 |
| 40 | 0.2163 | 0.363 | 0.0062 | 0.4128 | 0.0001 | 0.0016 | 1 |
| 41 | 0.311542 | 0.344026 | 0.311948 | 0.032179 | 0.000102 | 0.000203 | 1 |

| | | | | | | | |
|---|---|---|---|---|---|---|---|
| 42 | 0.509559 | 0.350722 | 0.087757 | 0.046268 | 0.005389 | 0.000305 | 1 |
| 43 | 0.5856 | 0.3532 | 0.0304 | 0.019 | 0.0114 | 0.0004 | 1 |
| 44 | 0.4078 | 0.4628 | 0.0615 | 0.0215 | 0.0459 | 0.0005 | 1 |
| 45 | 0.495193 | 0.40674 | 0.065074 | 0.03026 | 0.002631 | 0.000101 | 1 |
| 46 | 0.2083 | 0.4979 | 0.2456 | 0.0461 | 0.002 | 0.0001 | 1 |
| 47 | 0.442638 | 0.402088 | 0.079394 | 0.075379 | 0.0001 | 0.000401 | 1 |
| 48 | 0.1785 | 0.7837 | 0.0187 | 0.0177 | 0.001 | 0.0004 | 1 |
| 49 | 0.4418 | 0.3443 | 0.1297 | 0.0696 | 0.0145 | 0.0001 | 1 |
| 50 | 0.2148 | 0.6775 | 0.0535 | 0.0539 | 0.0002 | 0.0001 | 1 |
| 51 | 0.4357 | 0.5101 | 0.0059 | 0.0469 | 0.0013 | 0.0001 | 1 |
| 52 | 0.3475 | 0.4477 | 0.1783 | 0.0259 | 0.0004 | 0.0002 | 1 |
| 53 | 0.5992 | 0.3261 | 0.061 | 0.0135 | 0.0001 | 0.0001 | 1 |
| 54 | 0.431732 | 0.402402 | 0.056256 | 0.107307 | 0.002202 | 0.0001 | 1 |
| 55 | 0.4628 | 0.5218 | 0.0053 | 0.0077 | 0.0023 | 0.0001 | 1 |
| 56 | 0.5831 | 0.24 | 0.036 | 0.0813 | 0.0588 | 0.0008 | 1 |
| 57 | 0.5204 | 0.2411 | 0.0331 | 0.1817 | 0.0233 | 0.0004 | 1 |
| 58 | 0.5457 | 0.3468 | 0.0819 | 0.0252 | 0.0001 | 0.0003 | 1 |

Table S2. The total number of absolute bacterial abundances is almost the same.

| ID | A | B | C | D | E | F | Total |
|---|---|---|---|---|---|---|---|
| 1 | 4917 | 4120 | 807 | 127 | 22 | 4 | 9997 |
| 2 | 5956 | 3234 | 660 | 134 | 4 | 12 | 10000 |
| 3 | 4793 | 4603 | 19 | 330 | 239 | 16 | 10000 |
| 4 | 2355 | 4497 | 1637 | 645 | 214 | 1 | 9349 |
| 5 | 3284 | 6381 | 237 | 71 | 4 | 23 | 10000 |
| 6 | 4632 | 1747 | 2209 | 1358 | 49 | 5 | 10000 |
| 7 | 4429 | 3751 | 468 | 937 | 32 | 4 | 9621 |
| 8 | 3645 | 4677 | 99 | 630 | 948 | 1 | 10000 |
| 9 | 5096 | 3166 | 1003 | 526 | 1 | 5 | 9797 |
| 10 | 1263 | 7057 | 688 | 779 | 195 | 18 | 10000 |
| 11 | 4057 | 5207 | 559 | 109 | 4 | 2 | 9938 |
| 12 | 4295 | 4122 | 529 | 1028 | 18 | 8 | 10000 |
| 13 | 3620 | 5785 | 278 | 291 | 12 | 13 | 9999 |
| 14 | 3790 | 4992 | 387 | 291 | 537 | 3 | 10000 |
| 15 | 1935 | 6916 | 7 | 157 | 983 | 2 | 10000 |
| 16 | 4286 | 5322 | 96 | 216 | 79 | 1 | 10000 |
| 17 | 6134 | 3180 | 406 | 277 | 2 | 1 | 10000 |
| 18 | 2746 | 6937 | 87 | 217 | 1 | 12 | 10000 |
| 19 | 2833 | 6104 | 858 | 187 | 2 | 16 | 10000 |
| 20 | 3304 | 4132 | 483 | 372 | 1707 | 2 | 10000 |
| 21 | 4380 | 4067 | 766 | 773 | 13 | 1 | 10000 |
| 22 | 1912 | 7178 | 428 | 343 | 125 | 13 | 9999 |
| 23 | 1028 | 5888 | 2489 | 323 | 269 | 2 | 9999 |
| 24 | 2561 | 3959 | 361 | 506 | 2609 | 4 | 10000 |
| 25 | 2924 | 5479 | 220 | 426 | 949 | 2 | 10000 |

| | | | | | | | |
|---|---|---|---|---|---|---|---|
| 26 | 2608 | 6638 | 18 | 377 | 355 | 4 | 10000 |
| 27 | 2869 | 5377 | 1112 | 631 | 1 | 6 | 9996 |
| 28 | 4628 | 4616 | 395 | 331 | 29 | 1 | 10000 |
| 29 | 4907 | 3577 | 365 | 681 | 467 | 3 | 10000 |
| 30 | 4517 | 2474 | 453 | 422 | 2133 | 1 | 10000 |
| 31 | 3540 | 5470 | 379 | 378 | 184 | 49 | 10000 |
| 32 | 6250 | 1775 | 1209 | 48 | 35 | 39 | 9356 |
| 33 | 3343 | 5640 | 394 | 293 | 327 | 3 | 10000 |
| 34 | 5840 | 3482 | 179 | 97 | 5 | 12 | 9615 |
| 35 | 3850 | 4570 | 910 | 514 | 1 | 6 | 9851 |
| 36 | 4583 | 3956 | 766 | 228 | 466 | 1 | 10000 |
| 37 | 3969 | 5351 | 433 | 232 | 2 | 13 | 10000 |
| 38 | 3287 | 3145 | 3319 | 234 | 7 | 8 | 10000 |
| 39 | 3718 | 4801 | 266 | 1142 | 1 | 1 | 9929 |
| 40 | 2163 | 3630 | 62 | 4128 | 1 | 16 | 10000 |
| 41 | 3069 | 3389 | 3073 | 317 | 1 | 2 | 9851 |
| 42 | 5011 | 3449 | 863 | 455 | 53 | 3 | 9834 |
| 43 | 5856 | 3532 | 304 | 190 | 114 | 4 | 10000 |
| 44 | 4078 | 4628 | 615 | 215 | 459 | 5 | 10000 |
| 45 | 4893 | 4019 | 643 | 299 | 26 | 1 | 9881 |
| 46 | 2083 | 4979 | 2456 | 461 | 20 | 1 | 10000 |
| 47 | 4410 | 4006 | 791 | 751 | 1 | 4 | 9963 |
| 48 | 1785 | 7837 | 187 | 177 | 10 | 4 | 10000 |
| 49 | 4418 | 3443 | 1297 | 696 | 145 | 1 | 10000 |
| 50 | 2148 | 6775 | 535 | 539 | 2 | 1 | 10000 |
| 51 | 4357 | 5101 | 59 | 469 | 13 | 1 | 10000 |
| 52 | 3475 | 4477 | 1783 | 259 | 4 | 2 | 10000 |
| 53 | 5992 | 3261 | 610 | 135 | 1 | 1 | 10000 |
| 54 | 4313 | 4020 | 562 | 1072 | 22 | 1 | 9990 |
| 55 | 4628 | 5218 | 53 | 77 | 23 | 1 | 10000 |
| 56 | 5831 | 2400 | 360 | 813 | 588 | 8 | 10000 |
| 57 | 5204 | 2411 | 331 | 1817 | 233 | 4 | 10000 |
| 58 | 5457 | 3468 | 819 | 252 | 1 | 3 | 10000 |

Table S3. The total number of absolute bacterial abundances is different.

| ID | A | B | C | D | E | F | Total |
|---|---|---|---|---|---|---|---|
| 1 | 2459 | 2061 | 404 | 64 | 11 | 2 | 5000 |
| 2 | 5956 | 3234 | 660 | 134 | 4 | 12 | 10000 |
| 3 | 2876 | 2762 | 11 | 198 | 143 | 10 | 6000 |
| 4 | 2771 | 5291 | 1926 | 759 | 252 | 1 | 11000 |
| 5 | 16420 | 31905 | 1185 | 355 | 20 | 115 | 50000 |
| 6 | 27792 | 10482 | 13254 | 8148 | 294 | 30 | 60000 |
| 7 | 3683 | 3119 | 389 | 779 | 27 | 3 | 8000 |
| 8 | 2916 | 3742 | 79 | 504 | 758 | 1 | 8000 |
| 9 | 46814 | 29084 | 9214 | 4832 | 9 | 46 | 90000 |

| | | | | | | | |
|---|---|---|---|---|---|---|---|
| 10 | 3789 | 21171 | 2064 | 2337 | 585 | 54 | 30000 |
| 11 | 3266 | 4192 | 450 | 88 | 3 | 2 | 8000 |
| 12 | 17180 | 16488 | 2116 | 4112 | 72 | 32 | 40000 |
| 13 | 28963 | 46285 | 2224 | 2328 | 96 | 104 | 80000 |
| 14 | 18950 | 24960 | 1935 | 1455 | 2685 | 15 | 50000 |
| 15 | 5805 | 20748 | 21 | 471 | 2949 | 6 | 30000 |
| 16 | 85720 | 106440 | 1920 | 4320 | 1580 | 20 | 200000 |
| 17 | 4907 | 2544 | 325 | 222 | 2 | 1 | 8000 |
| 18 | 19222 | 48559 | 609 | 1519 | 7 | 84 | 70000 |
| 19 | 22664 | 48832 | 6864 | 1496 | 16 | 128 | 80000 |
| 20 | 29736 | 37188 | 4347 | 3348 | 15363 | 18 | 90000 |
| 21 | 17520 | 16268 | 3064 | 3092 | 52 | 4 | 40000 |
| 22 | 15298 | 57430 | 3424 | 2744 | 1000 | 104 | 80000 |
| 23 | 1028 | 5889 | 2489 | 323 | 269 | 2 | 10000 |
| 24 | 2305 | 3563 | 325 | 455 | 2348 | 4 | 9000 |
| 25 | 17544 | 32874 | 1320 | 2556 | 5694 | 12 | 60000 |
| 26 | 10432 | 26552 | 72 | 1508 | 1420 | 16 | 40000 |
| 27 | 14351 | 26896 | 5562 | 3156 | 5 | 30 | 50000 |
| 28 | 41652 | 41544 | 3555 | 2979 | 261 | 9 | 90000 |
| 29 | 19628 | 14308 | 1460 | 2724 | 1868 | 12 | 40000 |
| 30 | 9034 | 4948 | 906 | 844 | 4266 | 2 | 20000 |
| 31 | 2832 | 4376 | 303 | 302 | 147 | 39 | 8000 |
| 32 | 40081 | 11383 | 7753 | 308 | 224 | 250 | 60000 |
| 33 | 13372 | 22560 | 1576 | 1172 | 1308 | 12 | 40000 |
| 34 | 3644 | 2173 | 112 | 61 | 3 | 7 | 6000 |
| 35 | 4299 | 5103 | 1016 | 574 | 1 | 7 | 11000 |
| 36 | 22915 | 19780 | 3830 | 1140 | 2330 | 5 | 50000 |
| 37 | 23814 | 32106 | 2598 | 1392 | 12 | 78 | 60000 |
| 38 | 2630 | 2516 | 2655 | 187 | 6 | 6 | 8000 |
| 39 | 2996 | 3868 | 214 | 920 | 1 | 1 | 8000 |
| 40 | 19467 | 32670 | 558 | 37152 | 9 | 144 | 90000 |
| 41 | 9346 | 10321 | 9358 | 965 | 3 | 6 | 30000 |
| 42 | 4076 | 2806 | 702 | 370 | 43 | 2 | 8000 |
| 43 | 23424 | 14128 | 1216 | 760 | 456 | 16 | 40000 |
| 44 | 32624 | 37024 | 4920 | 1720 | 3672 | 40 | 80000 |
| 45 | 24760 | 20337 | 3254 | 1513 | 132 | 5 | 50000 |
| 46 | 6249 | 14937 | 7368 | 1383 | 60 | 3 | 30000 |
| 47 | 88528 | 80418 | 15879 | 15076 | 20 | 80 | 200000 |
| 48 | 1428 | 6270 | 150 | 142 | 8 | 3 | 8000 |
| 49 | 30926 | 24101 | 9079 | 4872 | 1015 | 7 | 70000 |
| 50 | 17184 | 54200 | 4280 | 4312 | 16 | 8 | 80000 |
| 51 | 39213 | 45909 | 531 | 4221 | 117 | 9 | 90000 |
| 52 | 13900 | 17908 | 7132 | 1036 | 16 | 8 | 40000 |
| 53 | 47936 | 26088 | 4880 | 1080 | 8 | 8 | 80000 |
| 54 | 4317 | 4024 | 563 | 1073 | 22 | 1 | 10000 |
| 55 | 4165 | 4696 | 48 | 69 | 21 | 1 | 9000 |

| | | | | | | | |
|---|---|---|---|---|---|---|---|
| 56 | 34986 | 14400 | 2160 | 4878 | 3528 | 48 | 60000 |
| 57 | 20816 | 9644 | 1324 | 7268 | 932 | 16 | 40000 |
| 58 | 27285 | 17340 | 4095 | 1260 | 5 | 15 | 50000 |
